# Annotation and cluster analysis of long noncoding RNA linked to male sex and estrogen in cancers

**DOI:** 10.1038/s41698-020-0110-5

**Published:** 2020-03-03

**Authors:** Shouping Liu, Weiwei Lai, Ying Shi, Na Liu, Lianlian Ouyang, Ziying Zhang, Ling Chen, Xiang Wang, Banglun Qian, Desheng Xiao, Qin Yan, Ya Cao, Shuang Liu, Yongguang Tao

**Affiliations:** 1 Department of Pathology, Xiangya Hospital, Key Laboratory of Carcinogenesis and Cancer Invasion (Ministry of Education) and School of Basic Medicine, Central South University, 410078 Hunan, China; 2 NHC Key Laboratory of Carcinogenesis (Central South University), Cancer Research Institute, Central South University, 410078 Changsha, Hunan China; 3 Department of Thoracic Surgery, Hunan Key Laboratory of Early Diagnosis and Precision Therapy in Lung Cancer, Second Xiangya Hospital, Central South University, 410011 Changsha, China; 4 Department of Oncology, Institute of Medical Sciences, National Clinical Research Center for Geriatric Disorders, Xiangya Hospital, Central South University, 410008 Changsha, Hunan China; 5 Department of Pathology, School of Basic Medicine and Xiangya Hospital, Central South University, 410008 Changsha, Hunan China; 6 Department of Pathology, Yale School of Medicine, New Haven, CT 06520 USA

**Keywords:** Cancer genomics, Computational biology and bioinformatics

## Abstract

The sex difference in cancer occurrence is a consistent finding in cancer epidemiology. Several solid tumors, including lung cancer, colorectal cancer, hepatic carcinoma, and renal carcinoma, are generally more common in males. Although sexual dimorphism is attributed to hormonal or behavioral differences, evidence for the function of lncRNA is lacking in sex-specific cancers. We show here that LINC00263 is one of the most dysregulated lncRNAs in lung adenocarcinomas and is upregulated in lung adenocarcinoma, colorectal cancer, and renal carcinoma, especially in male patients compared to females. LINC00263 functions as an oncogene by promoting translocation of p65 into the nucleus to activate the NF-κB-signaling pathway through interaction with IKKα in the cytoplasm. The expression of LINC00263 is strongly correlated with ESR1, and it is decreased after treatment with estrogen. Ligand-activated ER could inhibit the function of LINC00263 by inhibiting NF-κB from cytoplasmic translocation into the nucleus. The inhibitory effect of estrogen on LINC00263 indicates its differential expression in male and female patients. Our findings indicate that LINC00263 is linked to male sex and estrogen as an oncogene, and these findings might help in the exploration of the mechanisms of differential gene regulation in sex-specific cancers.

## Introduction

Sex differences in cancer constitute vital information that can be used to develop a causal hypothesis.^1^ Overall, males have a higher risk and worse prognosis than females in a wide varieties of cancer that are unrelated to reproductive function, such as cancers of the liver, lung, colon, and kidney.^2^ The generally higher cancer risk in the male population is attributed to diet and risky behaviors such as smoking and alcohol consumption.^3^ Additionally, the possible cellular and molecular basis of differences between the sexes in susceptibility to cancer include sex hormones and sex chromosomes.^3^ Both steroid and protein hormones are involved in sexual development and reproductive function.

Sex-related protein hormones, such as prolactin (PRL), luteinizing hormone (LH),^4^ follicle-stimulating hormone (FSH), and gonadotropin-releasing hormone (GNRH),^5^ are mainly implicated in cancers of reproductive tissues, including prostate, ovarian, and breast tissue.^6^ Growth hormone (GH) has been implicated in hepatocellular carcinoma,^7^ possibly because it undergoes pulsatile secretion into the plasma in males, and constant secretion in females.^8^ For the sex steroid hormones, much more abundant information exists on cancer development in nonreproductive organs relative to protein hormones. Estrogens and androgens can shuttle to the nucleus to affect gene expression,^9^ through affecting cancer stem cell self-renewal, the tumor microenvironment, the immune system, and the overall metabolic balance of an organism.

Lung cancer was the most frequently diagnosed cancer and the leading cause of cancer death among males in 2015. Among females, lung cancer was the leading cause of cancer death in more developed countries, and the second leading cause of cancer death in less developed countries.^2^ Lung cancer is divided into small cell lung cancer and non-small cell lung cancer (NSCLC), including lung adenocarcinoma (ADC), and squamous cell carcinoma (SCC).^10^ Epigenetic modifiers, such as long noncoding RNAs (lncRNAs), have important roles in the development and progression of NSCLC.^11–13^ LncRNAs could mediate important epigenetic regulation in a wide range of biological processes and diseases, because lncRNAs are expressed across the mammalian genome and contribute to the pervasive transcription phenomenon. They might display a tissue-specific and species-specific mode of expression.^14,15^

In our studies with ADC patient samples from TCGA, we found that there are 612 differentially expressed lncRNAs between tumor tissues and normal tissues, and one of the top five lncRNAs with the highest expression in the lung is LINC00263. There are few studies of LINC00263; herein, we investigated LINC00263-associated epigenetic regulation in cancers and successively performed GO term-enrichment analysis, KEGG pathway analysis, and GSEA analysis to discover the function in lung ADC. We also found that the expression of LINC00263 was sex-specific in several solid tumors and that estrogen inhibits the function of LINC00263.

## Results

### Frequent upregulation of LINC00263 in ADC

**Fig. 1:**
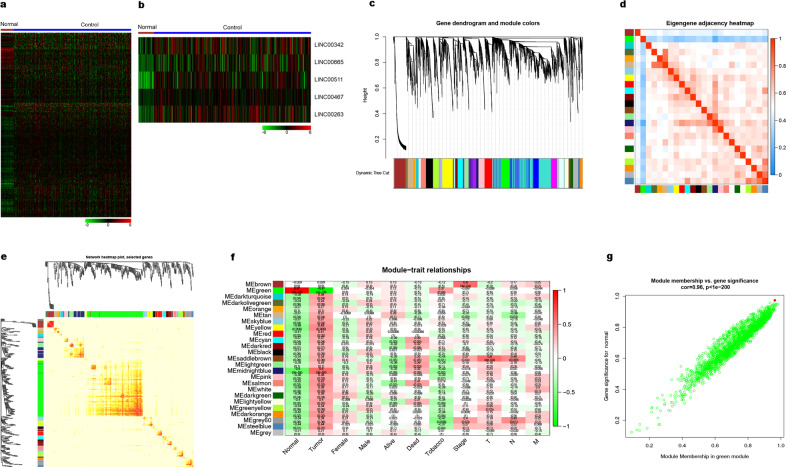
Bioinformatic analysis of lung ADC from the TCGA database. **a**, **b** Heatmap of the differentially expressed genes in lung ADC **a** 612 differentially expressed lncRNAs with 489 upregulated and 123 downregulated. **b** The top five lncRNAs with highest expression. **c** Differentially expressed gene clustering and module screening based on gene expression patterns of 30 lung ADC samples. Gene dendrogram obtained by clustering the dissimilarity based on consensus Topological Overlap with the corresponding module colors indicated by the color row. The top is the gene dendrogram and the bottom is the gene modules with different colors. A total of 25 modules were identified. **d**, **e** Heatmap of the correlation coefficient expressed between modules. Red represents high adjacency (positive correlation) and blue represents low adjacency (negative correlation). **f** Relationships of consensus module eigengenes and different traits such as normal, tumor, male, female, alive, dead, tobacco, stage, and TNM. Each row in the table corresponds to a module, and each column to a trait. Numbers in the table report the correlations of the corresponding module eigengenes and traits, with the *P*-values printed below the correlations in parentheses. The table is color coded by correlation according to the color legend. The intensity and direction of correlations are indicated on the right side of the heatmap (red, positively correlated; green, negatively correlated). **g** Analogous scatter plots for the green module. The gene significance for a tumor (*y*-axis) is strongly correlated with module membership in the green module (*x*-axis) cor = 0.96, *P* < 1e−200. The red dot represents LINC00263 (GS = 0.930878, MM = 0.92419).

To analyze the expression profiles and deregulation of lncRNAs in lung cancer, we interrogated the RNA-seq data from 59 normal tissue and 535 lung adenocarcinoma samples in TCGA. In this analysis, we found 12,583 differently expressed genes (FC > 1, *P* < 0.05, FDR < 0.001) and 612 differently expressed lncRNAs (Fig. 1a). Among these, we identified five lncRNAs as the most dysregulated in lung cancer, and we further addressed LINC00263 as one of the most dysregulated lncRNAs in lung adenocarcinomas (Fig. 1b).

To characterize the dynamic changes in lncRNA and mRNA expression, we clustered all their expression patterns (225 lncRNAs and 4875 mRNAs) by the WGCNA method.^16^ We chose the soft threshold power 8 to define the adjacency matrix based on the criterion of approximate scale-free topology with minimum module size 30 (Supplementary Fig. 1a). The module detection sensitivity is deepsplit2 and the cut height for the merging of modules is 0.2, which means that the modules whose eigengenes are correlated above 0.8 will be merged. We identified 25 main transcriptional modules, each represented by a characteristic expression pattern and by heat map graphing and eigengenes value graphing. Each colored row represents a color-coded module that contains a group of highly connected genes (Fig. 1c and Supplementary Fig. 1b). We found that some of the modules had similar expression profiles. Next, we performed a cluster analysis of the eigengenes connectivity among these 25 coexpressed modules (Fig. 1d, e). Tests of association between the phenotype variable of interest, such as normal–tumor status, sex, tumor stage, and the module eigengenes, were performed for each model. The results were summarized in a heatmap, and there were multiple modules related to normal–tumor states (Fig. 1f). The green module was the most significant module associated with normal–tumor status. We defined driver genes by gene significance (GS) in differential expression analysis and higher module membership (MM). We found LINC00263 (GS = 0.930878, MM = 0.92419) for the green module (Fig. 1g).

**Fig. 2:**
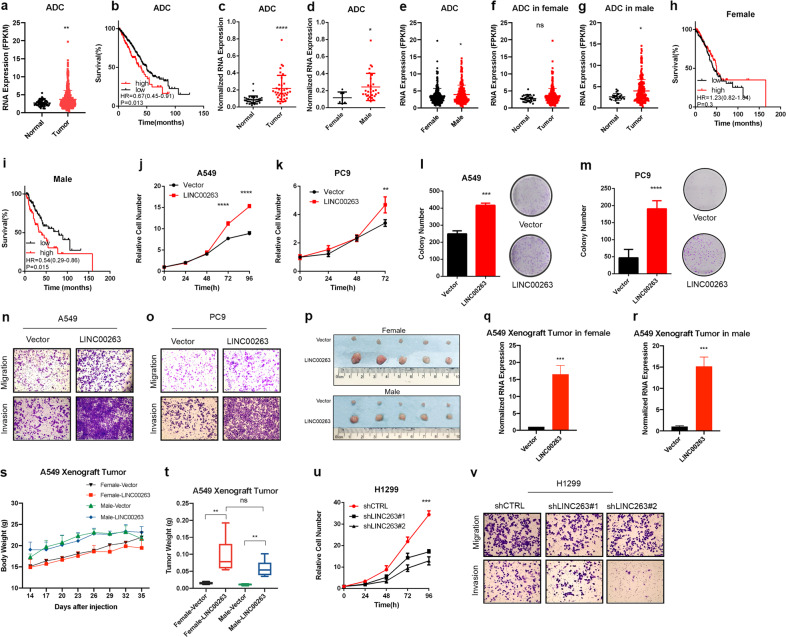
LINC00263 promotes cell growth, colony formation, invasion, and migration. **a** The expression of LINC00263 increased in 535 lung ADC tumor tissues compared to 59 normal tissues in the TCGA database. **b** Kaplan–Meier curves for overall survival rates associated with LINC00263 expression in lung ADC (low expression numbers = 366, high expression numbers = 125) HR = 0.67(0.45–0.91), *P* = 0.013. **c** RT-qPCR shows an increased expression of LINC00263 in 36 paired lung ADC relative to corresponding normal lung tissue samples *P* < 0.0001. **d** RT-qPCR verified the expression of LINC00263 in 29 male tumor tissue and 7 female tumor tissue (*P* = 0.0481). **e–g** Comparison of the expression of LINC00263 between 232 male tumor tissues and 265 female tumor tissue (*P* = 0.015) **e**, normal tissue and tumor tissue in females (*P* = 0.087) **f**, normal tissue and tumor tissue in males (*P* = 0.012) **g**. **h**, **i** Kaplan–Meier curves for overall survival rates associated with LINC00263 expression in female (low expression numbers = 140, high expression numbers = 129) **i** and males (low expression number = 110, high expression number = 52). **j**, **k** The MTS assay was used to assess cell viability in A549 **j** and PC9 **k** cells stably overexpressing LINC00263. The sex of A549 and PC9 was male. **l**, **m** Colony-formation assay of A549 **l** and PC9 **m** cells stably overexpressing LINC00263. **n**, **o** The results of cell migration and invasion. A549 **n** cells and PC9 **o** cells stably overexpressed LINC00263 have more cells migrated and invaded through the basement membrane compared to empty vector cells. **p–u** Nude mice after the injection of A549 stably expressing the control vector or LINC00263 expression plasmids are shown. Tumor formation was monitored at the indicated times; images **p**, weights **u** were recorded **v** MTS assay to assess cell viability in H1299 cells after the knockdown of LINC00263. The sex of H1299 was male. **w** H1299 cells after the knockdown of LINC00263 have fewer cells migrated and invaded through the basement membrane. Data are shown as the mean ± SEM; *n* ≥ 3 independent experiments, two-tailed Student’s *t*-test: ns nonsignificant (*P* > 0.05), *P < 0.05, **P < 0.01, ***P < 0.001.

Next, we evaluated LINC00263 transcription levels in ADC from TCGA. The data revealed that LINC00263 was significantly higher in lung ADC tissues than normal tissues (*P* < 0.01) (Fig. 2a). We also found that patients who had relatively higher levels of LINC00263 were associated with poor overall survival from lung ADC (low numbers = 366, high numbers = 125); this was assessed through a Kaplan–Meier analysis of a cohort of patients with lung ADC (Fig. 2b), indicating that LINC00263 might be a biomarker of lung ADC. To further validate that the expression level of LINC00263 might be higher in ADC, the expression levels of LINC00263 were detected in 36 lung ADC clinical samples, and we confirmed that LINC00263 was upregulated in lung ADC tumor tissues (Fig. 2c). These observations consistently showed that LINC00263 deregulation was a comment event in lung cancer and thus encouraged us to further study the significance of LINC00263 overexpression in lung cancer.

With the analysis of the relationship between LINC00263 and clinical features of lung ADC patients (Table 1), We found that the expression level of LINC00263 was not significantly correlated with patient age (*P* = 0.3005, *P* = 0.5470), tobacco exposure (*P* = 0.7351), tumor stage (*P* = 0.1634, *P* = 0.1133), differentiation (*P* = 0.9547, *P* = 0.4165), or tumor involvement (*P* = 0.8501, *P* = 0.396) but was related to sex (*P* = 0.0481); LINC00263 was highly expressed in male tumor tissues (Fig. 2d). We also noticed that the expression of LINC00263 in male lung ADC patients was significantly higher than that in female patients in the TCGA database (*P* < 0.0001) (Fig. 2e), whereas there was no such difference in normal lung tissue (*P* = 0.6595) (Supplementary Fig. 2a). Data from the GEO database also supports that LINC00263 was highly expressed in male tumor tissues (Supplementary Fig. 2b). We then separately analyzed the expression of LINC00263 in tumor tissues between normal tissues in female or male lung ADC patients. The phenomenon of LINC00263 as highly expressed in tumor tissues is not reflected in female patients (*P* = 0.08) (Fig. 2f, g). Finally, the high expression of LINC00263 had no significant effect on the survival prognosis of females (*P* = 0.3) (Fig. 2h); however, it was a significant disadvantage in male patients (*P* = 0.015) (Fig. 2i). Taken together, these findings indicate that the expression of LINC00263 is sex-specific in male lung ADC.

**Table 1:** LINC00263 expression level and clinical characteristics of lung cancer patients.

Factors	*n* (%)	Relative expression level (mean)	95% Confidence interval	*P*-value
*Gender*				
Male	29 (80.5%)	0.24	Reference	
Female	7 (19.5%)	0.11	0.001–0 .253	0.0481
*Age (year)*				
<50	8 (22.2%)	0.17	Reference	
50-60	20 (55.6%)	0.24	−0.074 to 0.219	0.3005
>60	8 (22.2%)	0.19	−0.053 to 0.096	0.5470
*Smoking*				
Yes	15 (41.7%)	0.23	Reference	
No	21 (58.3)	0.21	−0.088 to 0.125	0.7351
*Stage*				
I	11 (30.6%)	0.16	Reference	
II	12 (33.3%)	0.25	−0.042 to 0.231	0.1634
III	13 (36.1%)	0.23	−0.019 to 0.171	0.1133
*Differentiation*				
Low	15 (41.7%)	0.23	Reference	
Media	15 (41.7%)	0.23	−0.123 to 0.116	0.9547
High	6 (16.6%)	0.16	−0.106 to 0.245	0.4165
*Tumor involvement*				
None	11	0.22	Reference	
Pleura	20	0.23	−0.183 to 0.115	0.8501
Bronchus	5	0.09	−0.073 to 0.174	0.396

### LINC00263 promotes cell growth, colony formation, invasion, and migration

**Fig. 3:**
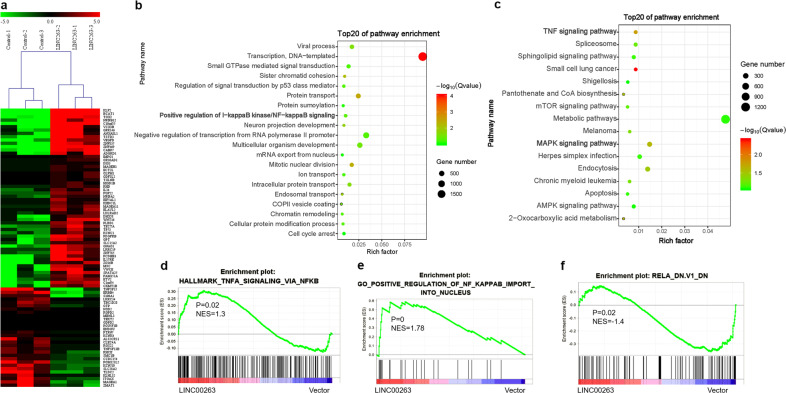
GO enrichment, KEGG, and GSEA analysis results for differentially expressed genes after the overexpression of LINC00263. **a** Heatmap of the differentially expressed genes after the overexpression of LINC00263 in A549 cells via RNA-seq, 58 mRNAs that were upregulated by two-fold (upregulated), and 26 mRNAs that were downregulated by two-fold. **b** GO-enrichment analysis results of differentially expressed genes with fold change>1. **c** KEGG-enrichment analysis of differentially expressed genes. **d–f** GSEA analysis of the whole transcriptome.

We first detected the expression levels of LINC00263 in a panel of lung ADC cells. RT-qPCR showed significantly increased LINC00263 in H1299 and H520 cell lines and reduced LINC00263 expression in A549, H358, and PC9 cell lines (Supplementary Fig. 2c). Therefore, we selected A549, PC9, and H1299 cells to determine the role of LINC00263. A549, PC9, and H1299 cell lines were derived from male lung adenocarcinoma patients. LINC00263 was successfully overexpressed in A549 and PC9 cell lines by full length RNA sequences (Supplementary Fig. 2d, e). We found that the overexpression of LINC00263 significantly enhanced the growth ability of A549 and PC9 in vitro (Fig. 2j, k). Moreover, stable expression of LINC00263 in A549 and PC9 cell lines significantly enhanced their colony formation abilities (Fig. 2l, m). Compared to the A549-Vector and PC9-Vector, the overexpression of LINC00263 was more vulnerable to invasion and migration (Fig. 3n, o). To address whether LINC00263 also has a role in lung cancer in vivo, and whether the sex of mice impact the function of LINC00263, we used a xenograft model in female and male mice. The injection of A549-LINC00263 showed that LINC00263 overexpression significantly increased tumor sizes, volumes, and weights after 35 days of growth compared with A549-Vector cells. But there was no such difference between females and males.

To further uncover the physiological role of LINC00263 in lung cancer, we performed the stable knockdown of LINC00263 in H1299 cells. LINC00263 was successfully knocked down in the H1299 cell line by using two different shRNA sequences (Supplementary Fig. 2f). The knockdown of LINC00263 in H1299 cells significantly slowed cell growth (Fig. 3v). Moreover, the knockdown of LINC00263 significantly inhibited invasion and migration (Fig. 3w).

Together, our results demonstrate that LINC00263 overexpression is linked to cell growth, colonization, invasion, and migration, suggesting that LINC00263 performs a critical oncogenic function in lung ADC progression.

### LINC00263 activates NF-κB-signaling pathway through interacting with IKKα

As we attempted to determine the molecular function of LINC00263 in lung ADC, we used RNA sequencing to analyze gene expression changes in the cells by comparing the stable expression of LINC00263 in A549 lung cells transfected with a control vector. After processing the data and averaging the replicates, compared to the control cells, we identified 58 genes that were increased by two-fold (upregulated) and 26 genes that were decreased by two-fold (downregulated) in the LINC00263 expression group (Fig. 3a). Moreover, analysis of significantly enriched GO terms indicated that these genes encode proteins concentrated mainly in the negative regulation of transcription from the RNA polymerase II promoter, cell proliferation and positive regulation of I-kappaB kinase/NF-κB signaling, and activation of NF-κB-inducing kinase activity (Fig. 3b). Furthermore, KEGG pathway analysis showed enrichment in metabolic pathways, the TNFα-signaling pathway and the mTOR-signaling pathway (Fig. 3c).

To further explore the mechanism of LINC00263 in lung ADC, we analyzed the transcriptome by GSEA. The overexpression of LINC00263 had a positive correlation with TNFA signaling via NF-κB and a positive regulation of NF-κB import into the nucleus (Fig. 3d, e), and a negative correlation with RELA knock down (Fig. 3f). Thus, these studies indicated that LINC00263 plays an important role in lung ADC by the PI3K/AKT/mTOR and NF-κB-signaling pathways.^17^

**Fig. 4:**
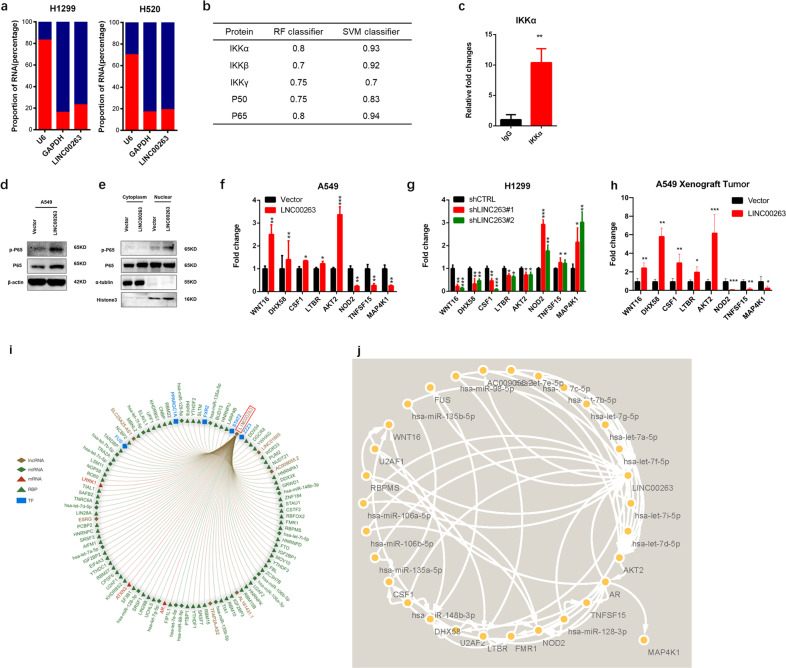
LINC00263 activates the NF-κB-signaling pathway through interaction with IKKα. **a** RT-qPCR analysis of LINC00263 expression levels in different subcellular fractions in H1299 and H520 cells and LINC00263 mainly localized in cytoplasm. **b** The prediction of the interaction between LINC00263 and IKKα, IKKβ, IKKγ, P50, and P65. **c** RIP assays show the association between IKKα and LINC00263. **d**, **e** LINC00263 overexpression promoted P65 transfer into the nucleus. **f–h** The expression of related genes was measured with RT-qPCR in A549 **e** cells stably overexpressing LINC00263 and H1299. **f** Cells stably transfected with two distinct target shRNA vectors and control cells and tumors in nude mice **h**. **i** miRNA, LncRNA, RBP, TF interacting with LINC00263. **j** Mechanism of LINC00263 with miRNA and target genes. Data are shown as the mean ± SEM; *n* ≥ 3 independent experiments, two-tailed Student’s *t*-test: ns nonsignificant (*P* > 0.05), *P < 0.05, **P < 0.01, ***P < 0.001.

Nuclear factor kappa-B (NF-κB) is a family of transcription-related factors that includes five genes: NF-κB1(P50/P105), NF-κB2 (P52/P100), RelA (P65), c-Rel, and RelB.^18^ In most types of cells, NF-κB dimers are predominantly cytoplasmic due to their interaction with the inhibitors of NF-κB (IκBs) and therefore remain transcriptionally inactive. Inhibitor of nuclear factor kappa-B kinase subunit alpha (IKKα), which is part of the IκB kinase (IKK) complex, plays an important role in regulating the NF-κB transcription factor as a noncranial pathway.^19^ The NF-κB proteins are key regulators of innate and adaptive immune responses that can accelerate cell proliferation, inhibit apoptosis, promote cell migration and invasion, and stimulate angiogenesis and metastasis.^20^ The cytoplasmic localization of LINC00263 was confirmed by subcellular fractionation analyses of H520 and H1299 cells (Fig. 4a), suggesting that LINC00263 may perform its biological functions in the cytoplasm. Then, we predicted the interaction between LINC00263 and the related proteins of the NF-κB-signaling pathway by the RNA–Protein Interaction Prediction,^21,22^ and determined that IKKα had the highest value (Fig. 4b). Therefore, RNA immunoprecipitation (RIP) assays using the anti-IKKα antibody confirmed that IKKα interacted with LINC00263 in PC9 cells (Fig. 4c).

To determine how LINC00263 participated in the NF-κB-signaling pathway, Western blotting showed that overexpressed LINC00263 promoted the expression of IKKα, IKKβ, and phosphorylated p65. It is worth noting that phosphorylated p65 is significantly increased in the nucleus (Fig. 4d, e). The classical NF-kB-signaling pathway is rapidly and transiently activated by pro-inflammatory cytokines, pathogen-associated molecular patterns (PAMPs), and damage-associated molecular patterns (DAMPs) that act via specific receptors and adaptor molecules that mainly target p50–p65 dimers. Moreover, the activation of NF-κB depends on the degradation of its specific inhibitors, the inhibitor of NF-κB (IκB) proteins, following their phosphorylation by the IKK complex.^23^ Our results also suggested that overexpressed LINC00263 could promote p65 translocation into the nucleus to activate the NF-κB-signaling pathway. Furthermore, the quantitative reverse transcription PCR (RT-qPCR) analysis confirmed that the trend of related genes with A549 and H1299 and tumors obtained from nude mice were consistent with RNA-Seq (Fig. 4f–h). To further understand the mechanism of LINC00263, we identified the top 100 mRNAs, lncRNAs, miRNAs, and transcription factors (TF) interacting with LINC00263 through the RAID database^24^ (Fig. 4i). LncRNA could directly adsorb or regulate the expression of miRNA, thereby indirectly regulating the expression of miRNA target genes. We analyzed the target genes of miRNAs that interact with LINC00263 and found that LINC00263 could act on AKT2, DHX58, NOD2, WNT16, TNFSF15, MAP4K1, and CSF1, which were involved in the NF-KB pathway by modulating has-let-7a-5p, has-miR-106a-5p, has-miR-135a-5p, has-miR-98-5p, and other miRNAs (Fig. 4j).

Taken together, these findings indicated that LINC00263 could promote p65 transfer into the nucleus through modulating the expression of miRNA and interacting with IKKα to activate the classical NF-κB-signaling pathway, which played an important role in the induction of genes involved in inflammation, cell proliferation and survival, the epithelial-to-mesenchymal transition and invasion, angiogenesis, and metastasis.

### LINC00263 links with cancers in males

**Fig. 5:**
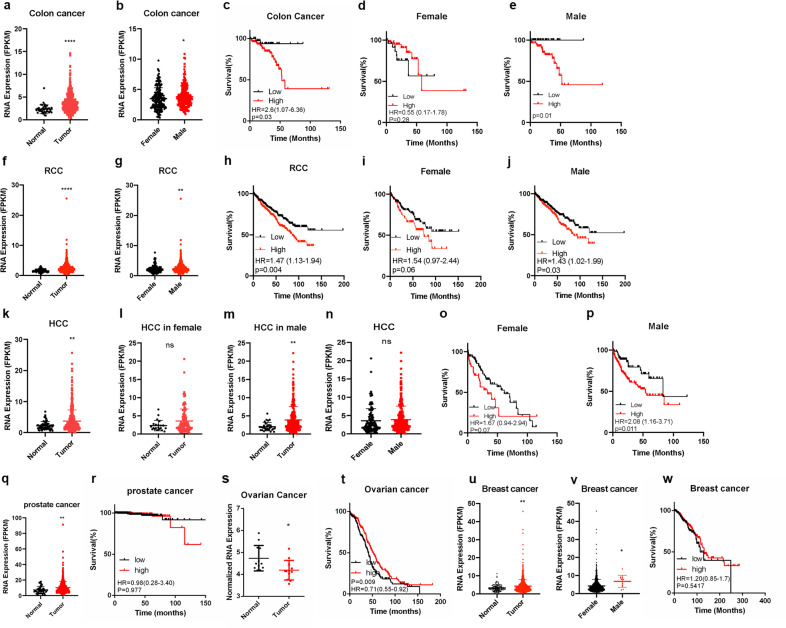
The expression of LINC00263 is sex-specific in cancers. **a** The expression of LINC00263 increased in 473 colon cancer tumor tissues compared to 41 normal tissue in the TCGA database (*P* < 0.0001). **b** Comparison of the expression of LINC00263 between 212 female tumor tissue and 235 male tumor tissues (*P* = 0.04). **c–e** Kaplan–Meier curves for overall survival rates associated with LINC00263 expression in colon cancers (low expression numbers = 140, high expression numbers = 129) **c** and females (low expression number = 28, high expression number = 47) **d** and males (low expression number = 32, high expression number = 58). **f** The expression of LINC00263 increased in 819 renal cell carcinoma tumor tissues compared to 113 normal tissue in the TCGA database (*P* < 0.0001). **g** Comparison of the expression of LINC00263 between 194 female tumor tissue and 396 male tumor tissues (*P* = 0.003). **h–j** Kaplan–Meier curves for overall survival rates associated with LINC00263 expression in renal cell carcinoma (low expression numbers = 418, high expression numbers = 399) **h** and females (low expression number = 155, high expression number = 107) **i** and males (low expression number = 298, high expression number = 257) **j**. **k** The expression of LINC00263 increased in 411 Hepatocellular carcinoma tumor tissues compared to 58 normal tissue in the TCGA database (*P* = 0.001). **l–n** Comparison of the expression of LINC00263 between normal tissues and tumor tissues in female (*P* = 0.08)(l) and in males (*P* = 0.006) **m** and between 120 female tumor tissues and 249 male tumor tissues (*P* = 0.38) **n**. **o**, **p** Kaplan–Meier curves for overall survival rates associated with LINC00263 expression in females (low expression number = 77, high expression number = 44) **o** and males (low expression number = 66, high expression number = 183) **p**. **q** The expression of LINC00263 increased in 499 prostate cancer tumor tissues compared to 52 normal tissue in the TCGA database (*P* = 0.005). **r** Kaplan–Meier curves for overall survival rates associated with LINC00263 expression in prostate cancer (low expression number = 247, high expression number = 248). **s** The expression of LINC00263 increased in 12 ovarian cancer normal tissues compared to 12 tumor tissue in the GEO database (GSE14407) (*P* = 0.01). **t** Kaplan–Meier curves for overall survival rates associated with LINC00263 expression in ovarian cancer (low expression number = 174, high expression number = 199). **u** The expression of LINC00263 increased in 1071 breast cancer tumor tissues compared to 98 normal tissue in the TCGA database (*P* = 0.007). **v** Comparison of the expression of LINC00263 between 1059 female tumor tissue and 12 male tumor tissues (*P* = 0.01). **w** Kaplan–Meier curves for overall survival rates associated with LINC00263 expression in breast cancer (low expression number = 354, high expression number = 723). Data are shown as the mean ± SEM; *n* ≥ 3 independent experiments, two-tailed Student’s *t*-test: ns nonsignificant (*P* > 0.05), *P < 0.05, **P < 0.01, ***P < 0.001.

Our findings indicate that the expression of LINC00263 is sex-specific in male lung ADC. Next, we wondered whether LINC00263 might be a common phenomenon in other sex-specific cancers. Herein, we evaluated LINC00263 transcription levels in squamous cell carcinoma, colorectal cancer, hepatic carcinoma, and renal carcinoma from TCGA. In squamous cell carcinoma, LINC00263 was higher in tumor tissues especially in male patients, but this phenomenon was not reflected in female patients. There was no significant difference between male patients and female patients and the high expression of LINC00263 had no significant effect on the survival prognosis (Supplementary Fig. 4a–f). In colorectal cancer, we showed that LINC00263 was highly expressed in male patients compared to female patients (Fig. 5a, b), whereas there was no such difference in normal colorectal tissue (Supplementary Fig. 4g–i) and the high expression of LINC00263 was a significant disadvantage in colorectal cancer (Fig. 5c). It was worth noting that the high expression of LINC00263 had no significant effect on the survival prognosis of females (Fig. 5d) but it has a significant disadvantage in male patients (Fig. 5e). Similarly, the expression level of LINC00263 was higher in male patients than female patients with renal cell carcinoma (Fig. 5f, g), and LINC00263 was also a disadvantage in male patients but had no significant effect in female patients (Fig. 5h–j). However, in hepatic carcinoma, LINC00263 was higher in tumor tissues than normal tissues but there was no significant difference between male patients and female patients (Fig. 5l). Male patients who had relatively higher levels of LINC00263 were associated with poor overall survival whereas there was no such difference in female patients (Fig. 5m–p). The same phenomenon was observed in the GEO database^25–27^ (Supplementary Fig. 4o–s).

Both prostate cancer and ovarian cancer are clearly related to sex. LINC00263 was highly expressed in tumor tissues in prostate cancer (*P* = 0.005) (Fig. 5q) but had no significant effect on survival prognosis (*P* = 0.977) (Fig. 5r), which may be related to the 10-year survival rate of prostate cancer of over 90%. The opposite result in ovarian cancer was seen, where the expression of LINC00263 was lower in tumors (*P* = 0.012) (Fig. 5s), and a high expression of LINC00263 was conducive to the prognosis of ovarian cancer (*P* = 0.009) (Fig. 5t). Similarly, LINC00263 was highly expressed in tumor tissues in breast cancer (*P* = 0.007) (Fig. 5u) and was also higher in male patients than female patients (Fig. 5v) but had no significant effect on survival prognosis (*P* = 0.5417) (Fig. 5w). In male breast cancer, the number of normal tissue is 1, and tumor tissues are 12 that the statistical analysis of the difference between normal and tumor tissues cannot be done. Collectively, LINC00263 is an example of sex-related lncRNA expression in cancer.

### LINC00263 links to estrogen

**Fig. 6:**
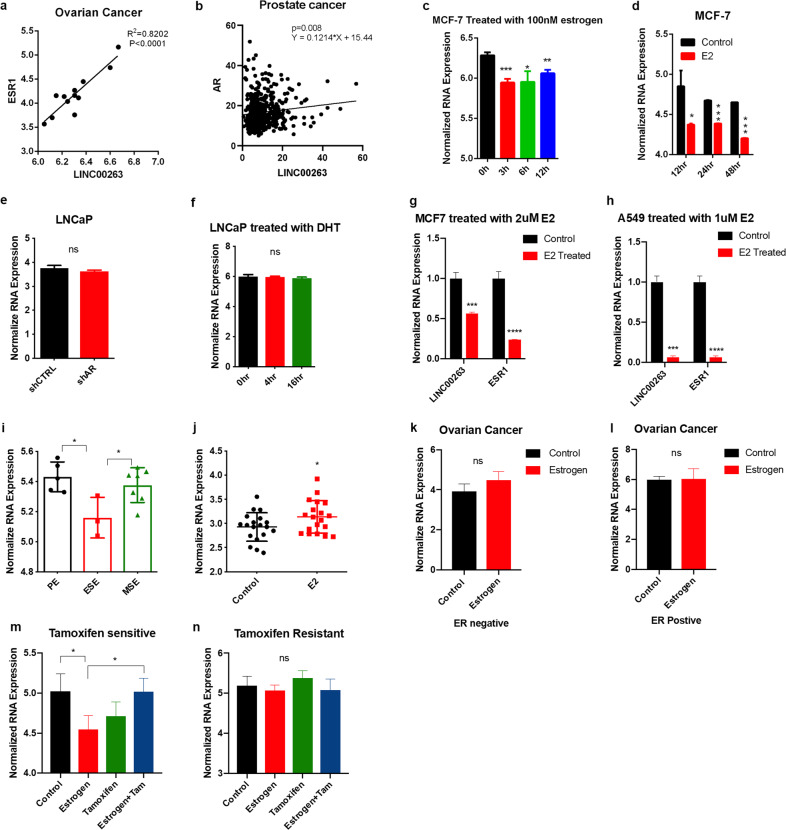
LINC00263 links with estrogen. **a** Correlation between ESR1 mRNA and LINC00263 LncRNA levels in ovarian cancer. Data from GEO database (GSE14407). **b** Correlation between AR mRNA and LINC00263 LncRNA levels in prostate cancer. Data from TCGA database. **c**, **d** The expression of LINC00263 in MCF-7 cells after treatment with 100 nM estrogen (GSE119087) **c** or estradiol (GSE11352) **d** from the GEO database. **e** The expression of LINC00263 in LNCaP cells after AR gene knock down. Data from GEO database (GSE11428). **f** The expression of LINC00263 in LNCaP cells after DHT treatment. Data from the GEO database (GSE7868). **g**, **h** RT-qPCR analysis was conducted to detect the level of LINC00263 after treatment with E2 in MCF-7 cells **g** and A549 cells **h**. **i** The expression of LINC00263 in normal women during the menstrual cycle. Data from the GEO database (GSE6364). **j** The expression of LINC00263 in the vagina of postmenopausal women after treatment with E2. Data from the GEO database (GSE11622). **k**, **l** the expression of LINC00263 in ER negative **k** or ER positive **l** ovarian cells after treatment with E2. Data from the GEO database (GSE22600). **m**, **n** The expression of LINC00263 in tamoxifen-sensitive **m** and tamoxifen-resistant **n** after treatment with E2 or tamoxifen or E2 and tamoxifen. Data from the GEO database (GSE26459). Data are shown as the mean ± SEM; *n* ≥ 3 independent experiments, two-tailed Student’s *t*-test: ns nonsignificant (*P* > 0.05), *P < 0.05, **P < 0.01, ***P < 0.001.

To clarify the potential mechanism that leads to the different expression of LINC00263 by sex, we evaluated the association of expression levels of LINC00263 with tobacco; the result was negative (Supplementary Fig. 4a, b). Then, we evaluated the association of the expression of LINC00263 with the gene *estrogen receptor 1* (ESR1) that encodes estrogen receptor α, *luteinizing hormone choriogonadotropin receptor* (LHCGR) that encodes LH,^4^ *follicle stimulating hormone subunit beta* (FSHB) that encodes FSH,^5^ *gonadotropin releasing hormone1* (GNRH1) that encodes GNRH,^5^ and *prolactin* (PRL) that encodes PRL.^6^ A strong correlation between LINC00263 and *ESR1* was found in ovarian cancer and there was a significant correlation between LINC00263 and *AR* in prostate cancer (Fig. 6a, b).^28^ However, there was no significant correlation with the other types (Supplementary Fig. 4c–f).

After a 100 nM estrogen treatment of the MCF-7 cell line, the expression of LINC00263 was significantly reduced (Fig. 6c),^29^ suggesting that LINC00263 had lower transcription levels in female patients because of the higher estrogen levels. In a similar way, in MCF-7 treated with estradiol (E2), which is the major female sex hormone, the expression of LINC00263 was significantly reduced (Fig. 6d).^30^ LNCaP cells are androgen-sensitive; however, with *AR* knockdown, the expression of LINC00263 did not change (Fig. 6e).^31^ Moreover, after treatment with dihydrotestosterone (DHT), an endogenous androgen sex steroid, the expression of LINC00263 also did not change with time (Fig. 6f).^32^ Thus, we mainly focused on the expression of LINC00263 associated with estrogen. We treated MCF-7 and A549 cells with E2 for 3 h and found that the expression of LINC00263 and ESR1 were decreased (Fig. 6g, h)

To further analyze the relationship between the expression of lINC00263 and estrogen, we analyzed the various phases of the menstrual cycle in normal women.^33^ At the proliferative phase (PE, d 8–14), estrogen secretion increased and peaked; at the early secretory phase (ESE, d 15-18), estrogen levels decreased; and in the midsecretory phase (MSE, d 19–23) estrogen levels rose slightly. Upon comparing the expression of LINC00263 in these three periods, it was found that the expression of LINC00263 in PE was the highest, followed by MSE, indicating that the expression of LINC00263 in the endometrium increased with the increase in estrogen level during the normal menstrual cycle (Fig. 6i).^33^ Similarly, compared to the control group, after treatment with E2, the expression of LINC00263 was increased in postmenopausal women (Fig. 6j) in the vaginal epithelium,^34^ indicating that LINC00263 is inversely affected by estrogen in breast cancer and the normal endometrium or vagina. We also found that after treatment with E2, the expression of LINC00263 in ER-positive or ER-negative ovarian cancer cells did not change (Fig. 6k, l).^35^

Tamoxifen is a competitive antagonist of E2 that binds to estrogen receptors in cells without stimulating transcription or weak effects. In tamoxifen-sensitive MCF-7 cells treated with E2, the expression of LINC00263 was decreased and then restored after treatment with tamoxifen and E2 (Fig. 6m). However, this phenomenon was not observed in tamoxifen-resistant MCF-7 cells (Fig. 6n),^36^ which indicated that ligand-activated ER could inhibit the function of LINC00263 in MCF-7 cells.

At the same time, we analyzed the difference in patients with breast cancer and ovarian cancer mutated in BRCA1 or BRCA2 or who were wild-type. The results suggested that the mechanism of LINC00263 development in breast cancer and ovarian cancer might be different (Supplementary Fig. 6a, b).^37^ The mechanism of LINC00263 in breast cancer was related to estrogen receptors and might be associated with BRCA gene mutations in ovarian cancer.

Upon menopause in females, production of estrogens by the ovaries stops and E2 levels decrease to very low levels. Breast cancer has a higher incidence, and aggressive behavior is higher in males and postmenopausal females compared with premenopausal females.^3^ We analyzed the expression of LINC00263 in breast cancer patients of different ages and found that LINC00263 was highly expressed in patients under 45 years old, but it was highly expressed in normal tissues from older patients (Supplementary Fig. 6c).^38^ But there was no significant difference in breast cancer survival after subdivided by age (Supplementary Fig. 6d, e). The reason might be that the high expression of LINC00263 had no significant effect on the survival prognosis in breast cancer. We divided the tumor samples into four subclasses (Luminal A, Luminal B, HER-2-enriched, and Triple negative), and the expression levels of LINC00263 and ESR1 were summarized in a heat map (Supplementary Fig. 6f).^39^ In the classification of breast cancer, ~30% of breast cancer patients had genetic alterations in the *HER-2* gene.^40^ *HER-2* positive cancers have been associated with poor overall survival and have been shown preclinically to enhance malignancy and the metastatic phenotype.^41^ Compared to ER-positive breast cancers, the NF-κB pathway is predominantly activated in ER-negative and ER-negative/HER-2 positive breast cancer.^42^ Interestingly, the expression of LINC00263 was higher in HER-2 positive breast cancer (Supplementary Fig. 6g). Then, we found a GEO database with the RELA gene activated in the SKR3 cell line, which was HER-2 positive, and the results showed that LINC00263 was upregulated by activating the NF-κB-signaling pathway (Supplementary Fig. 6h).^43^ NF-κB activity was increased in ER-positive MCF-7 cell lines that were resistant to Tamoxifen, and NF-κB activity was dependent on ER expression levels.^44^ We then compared the expression level of LINC00263 in a tamoxifen-sensitive MCF-7 with a tamoxifen-resistant MCF-7 cell line. The results showed that LINC00263 was highly expressed in drug-resistant cells (Supplementary Fig. 6i).^28^

**Fig. 7:**
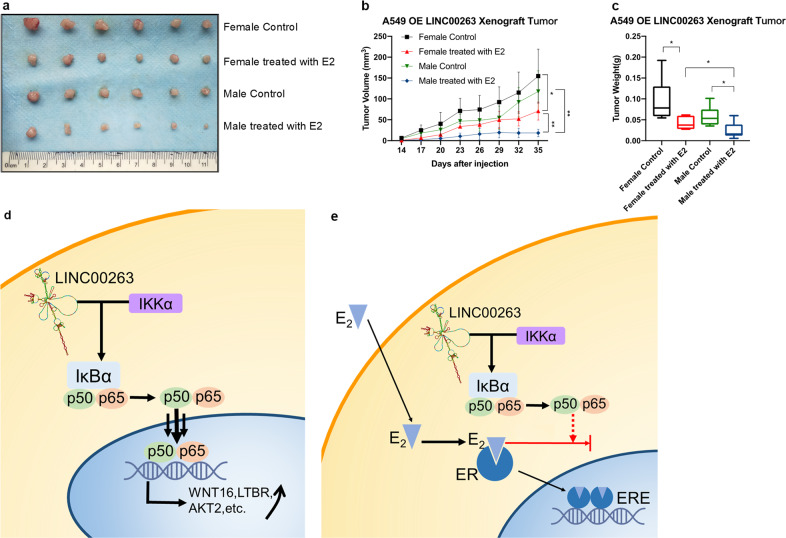
The xenograft model of A549-LINC00263 and schematic diagram predicting the role of LINC00263. **a–c** Nude mice after the injection of A549 stably expressing the LINC00263 expression are shown. Tumor formation was monitored at the indicated times; images **a**, volume **b** and weights **c** were recorded (*n* = 8). Data are shown as the mean ± SEM; *n* ≥ 3 independent experiments, two-tailed Student’s *t*-test: ns nonsignificant (*P* > 0.05), *P < 0.05, **P < 0.01, ***P < 0.001. **d** LINC00263 could promote p65 translocation into the nucleus to activate the NF-κB pathway signaling through interaction with IKKα. Estrogen binding with estrogen receptor to inhibit p65 entry into the nucleus that can inhibit the function of LINC00263.

Our studies indicated that the expression of LINC00263 was related to estrogen in cancers. To address whether LINC00263 is also affected by estrogen in vivo, we used a xenograft model. With the injection of A549-Vector, E2 treatment had no significant effect on tumor weight in mice (Supplementary Fig. 7). However, with the injection of A549-LINC00263 cells, E2 treatment significantly decreased tumor sizes, volumes, and weights compared with the controls. We also noticed that after treatment with E2, the tumor sizes, volumes, and weights of male nude mice were smaller than that of female mice, while this phenomenon was not observed in DMSO-treated mice. The whole-body weights all remained unchanged (Fig. 7a–c). Together, our results demonstrated that estrogen could affect the function of LINC00263 in cancers; estrogen played a protective role, which was more prominent in male nude mice supplemented with estrogen.

Collectively, these results demonstrate that LINC00263 is linked to estrogen. Ligand-activated ER could inhibit the function of LINC00263 by inhibiting the translocation of NF-κB from the cytoplasm into the nucleus (Fig. 7d). The inhibitory effect of estrogen on LINC00263 indicates its differential expression in male and female cancer patients.

## Discussion

The incidence of many types of cancer arising in organs with nonreproductive functions is significantly higher in male populations than in female populations, with associated differences in survival.^45^ Except for occupational and behavioral factors,^46^ cellular and molecular differences are also important. A recent analytic approach based on the propensity score revealed molecular differences in sex-specific cancers due to mutations, DNA methylation, transcripts, and protein expression.^47^ Except for sex hormones and sex chromosomes,^48^ the Alu editing index (AEI) associated with GBM patient overall survival in a sex-specific manner.^49^ However, there are still no studies showing that lncRNA is associated with sex-specific cancers. Here, we presented a bioinformatics analysis of the TCGA and GEO data to guide future research in sexual dimorphism.

We demonstrate that LINC00263 was overexpressed in several cancers, including lung adenocarcinoma, squamous cell carcinoma, colorectal cancer, renal cell carcinoma, hepatic carcinoma, breast cancer, and prostate cancer based on the analysis of gene expression data. Except for hepatic carcinoma and squamous cell carcinoma, the expression of LINC00263 was high in male patients. LINC00263 is located at 10q24.31, and it is worth noting that the expression of LINC00263 is significantly negatively correlated with *X-inactive specific transcript* (XIST) (Supplementary Fig. 8), which is required for transcription silencing of one X chromosome during development in female mammals.^50^ This might be one of the possible mechanisms for the different expression level of LINC00263 in different sexes. High expression of LINC00263 was a disadvantage in the survival prognosis of lung adenocarcinoma, renal and clear cell carcinomas, colorectal cancer and hepatic carcinoma, but it was a favorable factor in ovarian cancer and had no significant effect in prostate cancer and breast cancer. Our results suggest that LINC00263 is higher in male patients than females in sex-specific cancers and it might be used as an assessment of survival prognosis in lung adenocarcinoma, renal cell carcinoma, colorectal cancer, and hepatic carcinoma.

Moreover, our findings provide evidence that LINC00263 has an important role in lung adenocarcinoma. LINC00263 overexpression significantly promotes cancer progression. By significance analysis, including GO term-enrichment analysis, KEGG pathway analysis and GSEA analysis, LINC00263 might affect cancer progression through the PI3K/AKT/mTOR and NF-κB pathways. Here, we present strong evidence showing that LINC00263 overexpression could promote the expression of phosphorylated p65 and promote the phosphorylated p65 transfer into the nucleus to activate the NF-κB-signaling pathway.

A large number of research results indicated that ER could inhibit or promote NF-κB activation signaling pathways, including upstream segments in the cytoplasm and downstream segments in the nucleus.^51–54^ Ligand-activated ER might inhibit transcriptional activity by inhibiting NF-κB from translocation into the nucleus without interfering with upstream IKK and IκB.^55^ We hypothesized that estrogen bound with ER could inhibit p65 import into the nucleus to affect the expression level of LINC00263. In the treatment of breast cancer, Tamoxifen, which is a selective estrogen-receptor modulator (SERM),^56^ is currently used for the treatment of both early and advanced estrogen receptor-positive breast cancer in pre- and post-menopausal women.^57^ Unfortunately, up to half of ERα-positive tumors have intrinsic or acquired endocrine therapy resistance.^58^

Previous studies claimed that E2 inhibits NF-κB-mediated gene transcription,^59,60^ and decreased activity of p65 induced by E2 is due to the cytoplasmic sequestration of this transcription factor.^55^ Our results showed that LINC00263 is expressed at lower levels in female patients than in male patients. The expression of LINC00263 is higher in ER-negative and HER-2 positive breast cancers, where NF-κB is predominantly activated. With E2 treatment, the expression of LINC00263 in the MCF-7 cell line decreased, which suggested that E2 has an inhibitory effect on LINC00263. We suspect that LINC00263 might promote the p65 transfer to the nucleus prevented by E2. It is of interest to note that NF-κB is also activated in MCF-7 cell lines with Tamoxifen resistance, and the expression of LINC00263 is higher in Tamoxifen-resistant MCF-7 than Tamoxifen sensitive MCF-7 cell lines. Approximately 35% of ER-positive breast cancers that initially respond to the adjuvant therapy with tamoxifen will eventually relapse with endocrine resistance within 15 years.^61^ There are several studies showing that surgical resection of primary cancer and chemotherapy induce DNA damage and genotoxic stress, which evoke a subsequent inflammatory response that results in NF-κB activation and the upregulation of cytokines and chemokines, which promote therapy resistance and tumor recurrence.^62^ Interestingly, the tamoxifen resistance in the treatment of breast cancer might be related to the activation of NF-κB. These results provide new insights into the study of tamoxifen resistance and remind us that the expression level of LINC00263 could be a new biomarker to detect whether endocrine therapy is effective.

Our study illustrates that LINC00263 functions as an oncogene to facilitate tumor cell proliferation, and its overexpression in male patients in several solid tumors because of estrogen could inhibit the function of LINC00263. The expression of LINC00263 is significantly different between males and females, and it might be a tool to explore the mechanisms of differential gene regulation in sex-specific cancers. LINC00263 overexpression could promote p65 transfer into the nucleus through interaction with IKKα to activate NF-κB, which might be related to therapy resistance and tumor recurrence. Therefore, the high level of LINC00263 is expected to be one of the indicators of tamoxifen resistance in breast cancer, and the relationship between estrogen and the NF-κB-signaling pathway is expected to become a breakthrough in sex-specific cancers.

## Methods

### TCGA data description

The public available TCGA datasets were directly downloaded from the TCGA Data Portal at https://tcga-data.nci.nih.gov/tcga/. The detailed information for the TCGA data structures can be reviewed at https://tcga-data.nci.nih.gov/tcga/tcgaDataType.jsp. The detailed information for the microarray and RNA-Seq experiments, protocols, and software used can be found at the TCGA Data Portal at https://tcga-data.nci.nih.gov/tcga/. As described, RNA-seq data for lung carcinoma, colorectal cancer, hepatic carcinoma, and renal carcinoma were obtained from the Genomic Data Commons Data Portal.^63^ Due to the retrospective nature of this study using only publicly available data, ethics approval was not required.

### Acquisition of microarray data

The Gene Expression Omnibus (GEO, http://www.ncbi.nlm.nih.gov/geo/) database at the National Center for Biotechnology Information (NCBI) is used to store curated gene expression datasets, original series, and platform records. The statistical software R (version 3.5.1, https://www.r-project.org/) and Bioconductor packages (http://www.bioconductor.org/) were applied to significance analysis of differentially expressed genes. The integrative algorithm “gcRMA” was chosen for preprocessing of microarray data.^64^ An empirical Bayes method was used to select significant DEGs based on the “limma” package of Bioconductor.^65^ Differentially expressed genes were considered to have statistical significance and to achieve significant enrichment. Due to the retrospective nature of this study using only publicly available data, ethics approval was not required.

### Weighted correlation network analysis (WGCNA)

As a systems biology method, gene coexpression network analysis was performed using the WGCNA package to describe the correlation of gene expression pattern and to screen highly correlated gene modules, holding promise for finding candidate biomarkers, and drug targets.^66^ In this coexpression network, nodes represented differentially expressed genes and the correlation of gene expression pattern was defined as the connectivity degree among genes.^67^ In brief, excessive missing values and outlier microarray samples were first detected according to a differentially expressed gene expression matrix. The soft thresholding power was determined by analysis of network topology. Gene coexpression similarity and adjacency were successively calculated using the soft thresholding power. Then, the adjacency was transformed into a topological overlap matrix (TOM). Finally, hierarchical clustering was conducted using TOM and the dynamic tree cut algorithm was applied to module screening, after which we performed GO-enrichment analysis on gene modules to characterize modules related to ADC.

### GO term and KEGG pathway-enrichment analysis

Biological significance of differentially expressed genes was explored by GO term-enrichment analysis and KEGG pathway-enrichment analysis by DAVID Bioinformatics Resources 6.8 (https://david.ncifcrf.gov/home.jsp).^68^

### Cell culture, chemicals, plasmids, and siRNAs

Lung cancer cell line A549 (ATCC: CCL-185^TM^) was obtained from American Type Culture Collection. PC9 and H1299 cells were obtained from the Cancer Research Institute of Central South University. The sex of A549, PC9, and H1299 was male. PC9 and H1299 were cultured in Roswell Park Memorial Institute 1640 Media (RPMI 1640; Gibco, USA) supplemented with 10% fetal bovine serum (FBS). A549 cells were cultured in Dulbecco’s Modified Eagle Medium/Nutrient Mixture F-12 (DMEM/F12) 1:1 (HyClone), 293T cells were cultured in Dulbecco’s Modified Eagle’s medium (Gibco), and the other cell lines were cultured in RPMI 1640 (Gibco). All media were supplemented with 10% (V/V) FBS. All cell lines were maintained at 37 °C with 5% CO_2_. All cell lines tested negative for mycoplasma contamination and were passaged <10 times after the initial revival from frozen stocks. All cell lines were authenticated before use by short tandem repeat profiling. The LINC00263 coding region was sub‐cloned into the retroviral vector pLVX‐EF1α‐IRES‐Puro (Clontech, Mountain View, CA, USA) purchased from Beijing Genomics Institute (Beijing, China). LINC00263 small hairpin RNA (shRNA) vectors (GV248, LINC00263‐shRNA 1–3, and control shRNA) were purchased from GeneChem (Shanghai, China). The transfection of plasmids was performed using Lipofectamine^®^ 2000, according to the manufacturer’s protocol, and stably expressing colonies were selected using 2 mg/ml puromycin.

### Cell proliferation and colony formation assays

The cell proliferation assay was performed with a CellTiter 96 AQueous One Solution Cell Proliferation Assay (MTS,3-(4,5-dimethylthiazol-2-yl)-5-(3-carboxymethoxyphenyl)-2-(4-sulfophenyl)-2H-tetrazolium) as per the manufacturer’s protocol. First, 200 cells were cultured in a 96-well plate. The OD450 was measured 1 h after adding MTS. For the cell colony formation assay, ~200 cells were seeded into the wells of six-well plates and cultured in media. After 2 weeks, cells were treated with methanol and stained with 0.1% crystal violet. The number of visible colonies was counted using ImageJ software and colonies of >0.05 mm in diameter were scored.^69^

### RT-qPCR assays

Total RNA was extracted from ADC cells or tissues using TRIZOL Reagent (Invitrogen), following the manufacturer’s protocol. Cytoplasmic RNA and nuclear RNA were separated and purified using RNAiso Blood (Takara, Dalian, China), following the manufacturer’s instructions. Real-time PCR was performed using the detection system ABI 7500 with FastStart Universal SYBR Green Master (ROX).

The following primers for RT‐qPCR were purchased from Sangon Biotech (Shanghai, China):

LINC00263: forward, 5′-TCGGATAGGAGTGTCAGG-3′, and reverse, 5′-TTCAGTTGCTTCAGGTCAT-3′

β‐actin: forward, 5′‐CACCATTGGCAATGAGCGGTTC‐3′, and reverse, 5′‐AGGTCTTTGCGGATGTCCACGT‐3′;

GAPDH: forward, 5′‐GTCTCCTCTGACTTCAACAGCG‐3′, and reverse, 5′‐ACCACCCTGTTGCTGTAGCCAA‐3′;

U6: forward, 5′‐CTCGCTTCGGCAGCACA‐3′, and reverse, 5′‐AACGCTTCACGAATTTGCGT‐3′.

The relative fold changes in messenger RNA expression were calculated using the 2−ΔΔCt method. Calculation of the ΔCt value used the following formula: ΔCt(target) = Ct(target) − Ct(β‐actin).

### Cell migration and invasion assays

Cells were collected 48 h post‐transfection; 5 × 10^4^ (for migration assays) or 1 × 10^5^ (for invasion assays) cells in serum‐free medium were placed into the upper chamber of an insert (8 μm pore size; Falcon, San Jose, CA, USA). Matrigel Matrix (Falcon) diluted nine times was added to the upper chamber of an insert before the serum‐free medium. Medium containing 10% FBS was added to the lower chamber. After 24 h of incubation, the cells remaining on the upper membrane were removed with cotton wool; cells that had migrated or invaded through the membrane were stained with methanol and 0.1% crystal violet and imaged. Experiments were independently repeated three times.

### Western blot assay

Cells stably overexpressing LINC00263 were lysed by RIPA buffer with a protease inhibitor cocktail (Roche). A protein assay kit (Bio‐Rad) was used to determine the concentration of protein. Protein lysates weighing 50 μg were electrophoresed on 12% sodium dodecyl sulfate–polyacrylamide gels, transferred onto 0.45‐μm polyvinylidene fluoride membranes (Millipore, Billerica, MA, USA), and then incubated with Antibody Sampler Kit (Cell Signaling, Danvers, MA, USA). P65 antibody (A11155, AB clonal), p-P65 antibody (AP0124, AB clonal), β‐Actin antibody (mouse mAb, Sigma-Aldrich, Cat.A2228-100 μl). Pierce ECL Western Blotting Substrate (Thermo Scientific) was used to detect the bands and quantify the intensity. β‐Actin antibody was used as a control.^70^

### RIP assays

First, 4 × 10^7^ cells were crosslinked for 10 min at 37 °C using 0.3% formaldehyde in the medium. Cells were washed with cold 1 × PBS twice and then lysed in RIPA buffer (50 mM Tris pH 7.4, 150 mM NaCl, 1 mM EDTA, 0.1% SDS, 1% NP-40, 0.5% sodium deoxycholate, and 0.5 mM dithiothreitol with RNase inhibitor and protease inhibitor cocktail) followed by the mechanical shearing of chromatin using a Dounce homogenizer for 15–20 strokes. Immunoprecipitation was performed by incubating protein A/G magnetic beads with precleared lysates. Lysates were incubated with 2.0 μg of mouse monoclonal anti-IKKα (Santa Cruz, sc-71290) with an equivalent amount of normal mouse IgG at 4 °C for 16 h. The RNA/antibody complex was then precipitated by incubation with protein A/G magnetic beads. After three rounds of rinsing with ice-cold 1 × PBS, the RNA samples were extracted with Trizol reagent and detected by RT-qPCR.^71^

### Survival analysis

An independent *t*-test was performed to calculate the difference between groups. Kaplan–Meier survival analysis was performed to compare the survival distribution between different groups by using GraphPad Prism software.^72^ A plot of the Kaplan–Meier analysis^73^ with appropriate sample size provides the information on the length of survival, median survival time of the distinct sample populations, and significance of the difference between the survival curves.

### Nude mice and study approval

Athymic nude mice (eight female nude mice and eight male nude mice, aged 4 weeks) were divided into two groups (four female nude mice and four male nude mice for each group) and injected in the armpit with A549-Vector or A549-LINC00263 cells (4 × 10^6^).^74^ Four nude mice receiving A549-Vector (two males and two females) were intraperitoneally injected with DMSO, and the other four nude mice were injected with E2 (1.5 mg/mouse) each week. Eight nude mice receiving A549-LINC00263 underwent the same processing. We used β-E2 (Sigma, E2758-250MG). All procedures for animal studies were approved by the Institutional Animal Care and Use Committee of the Central South University of Xiangya School of Medicine and conformed to the legal mandates and federal guidelines for the care and maintenance of laboratory animals. SCID mice (Hunan SJA Laboratory Animal Co. Ltd.) were injected with the indicated cells. Injected mice were imaged from both the dorsal and ventral sides every 3 days. Data were analyzed using Student’s *t*-test; a *P* value < 0.05 was considered significant.

### Statistical analysis

All statistical analyses were performed using the GraphPad Prism 8.0 software. The data are presented as the mean ± SEM from multiple individual experiments each performed in triplicate and each experiment was repeated at least three times. Student’s *t*-test (two-tailed) was applied to compare differences between two groups and ns is non-significant (*P* > 0.05), *P < 0.05, **P < 0.01, ***P < 0.001. The relationships between LINC00263 and ESR1 and XIST expression levels were determined by using the Spearman’s correlation coefficient (*R*), where the *P*-value reflects the result of a *t*-test in which the null hypothesis is that the correlation between the variables is equal to zero.

## Supplementary information

Supplementary Materials

Reporting Sum

## Data Availability

The data that support the findings of this study are available from the corresponding author upon reasonable request. Datasets used for research are publicly available on the GDC data portal under the TCGA-LUAD, TCGA-LUSC, TCGA-BRCA, TCGA-KIRC, TCGA-KIRP, TCGA-KICH, TCGA-COAD, TCGA-LIHC, TCGA-PRAD projects (https://portal.gdc.cancer.gov). GEO datasets used for research are listed in Supplementary Table 1.

## Code availability

Relevant code is deposited into Github: https://github.com/lsp13/TCGA-analyze and available for everyone. R 3.5.1 was used for data analyze.
