# Supplementary material for: Annotation and cluster analysis of long noncoding RNA linked to male sex and estrogen in cancers
Source: NPJ Precis Oncol. 2020 Mar 3;4:5. doi: 10.1038/s41698-020-0110-5 (PMC7054536; doi:10.1038/s41698-020-0110-5)
Supplement: Supplementary file 1 — Supplementary Materials [file 41698_2020_110_MOESM1_ESM.pdf]

Supplementary Information for

Annotation and cluster analysis of long noncoding RNA linked to male sex and estrogen in cancers

Shouping Liu [1,2], Weiwei Lai [1,2], Ying Shi [1,2], Na Liu [1,2], Lianlian Ouyang [1,2], Ziying Zhang [1,2], Ling Chen [1,2], Ya Cao [1], Xiang Wang [3], Banglun Qian [3], Desheng Xiao [1,4,5], Qin Yan [6,] Shuang Liu [1, 4*], Yongguang Tao [1,2,3,*]

Correspondence to: taoyong@csu.edu.cn, shuangliu2016@csu.edu.cn

This PDF file includes:
Supplementary Figure 1-8, Supplementary Table 1.

Supplementary Figure 1

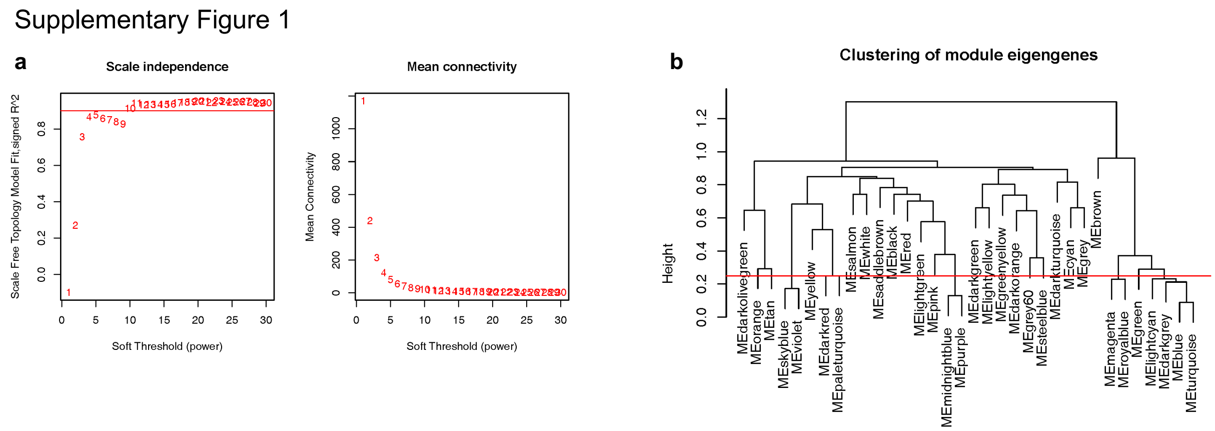

Supplementary Figure 1. Analysis of network topology for various soft-thresholding powers and Sample clustering to detect outliers. **a** Network topology for different soft-thresholding powers. Numbers in the plots indicate the corresponding soft thresholding powers. The approximate scale-free topology can be attained at the soft-thresholding power of 8. **b** Dendrogram of consensus module eigengenes obtained by WGCNA on the consensus correlation. The red line is the merging threshold, and groups of eigengenes below the threshold represent modules whose expression profiles should be merged due to their similarity.

Supplementary Figure 2

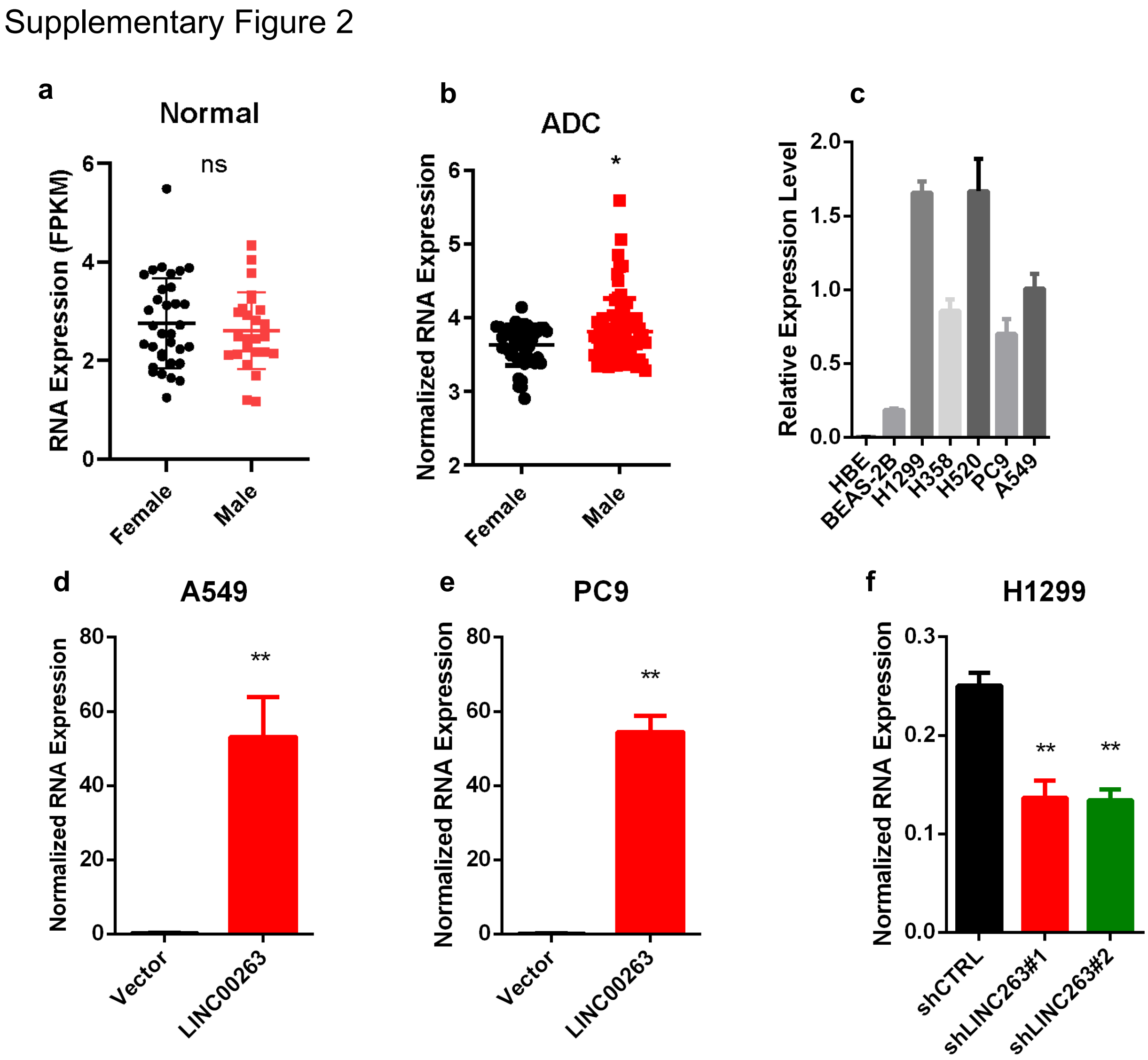

Supplementary Figure 2. The expression of LINC00263 in ADC and lung cancer cells and the detection of LINC00263 over-expression or knockdown efficient. **a** the expression of LINC00263 from ADC patient normal tissues in males and females. Data from the TCGA database. **b** the expression of LINC00263 is higher in 69 male patients than 44 female patients (P=0.0167). Data from the GEO database (GSE50081). **c** RT-qPCR analysis was conducted to detect the level of LINC00263 in HBE, BEAS-2B, H1299, H358, H520, PC9 and A549 cell lines. **d, e** A RT-qPCR analysis was conducted to detect the level of LINC00263 in A549 (**d**) and PC9 (**e**) cells stably

overexpressing LINC00263. **f** RT-qPCR analysis to detect the level of LINC00263 in H1299 cells stably transfected with two distinct target shRNA vectors and control cells. Data are shown as the mean ± SEM; n ≥ 3 independent experiments, two-tailed Student's t-test: ns nonsignificant ($P > 0.05$), $^{*}P < 0.05$, $^{**}P < 0.01$, $^{***}P < 0.001$

Supplementary Figure 3

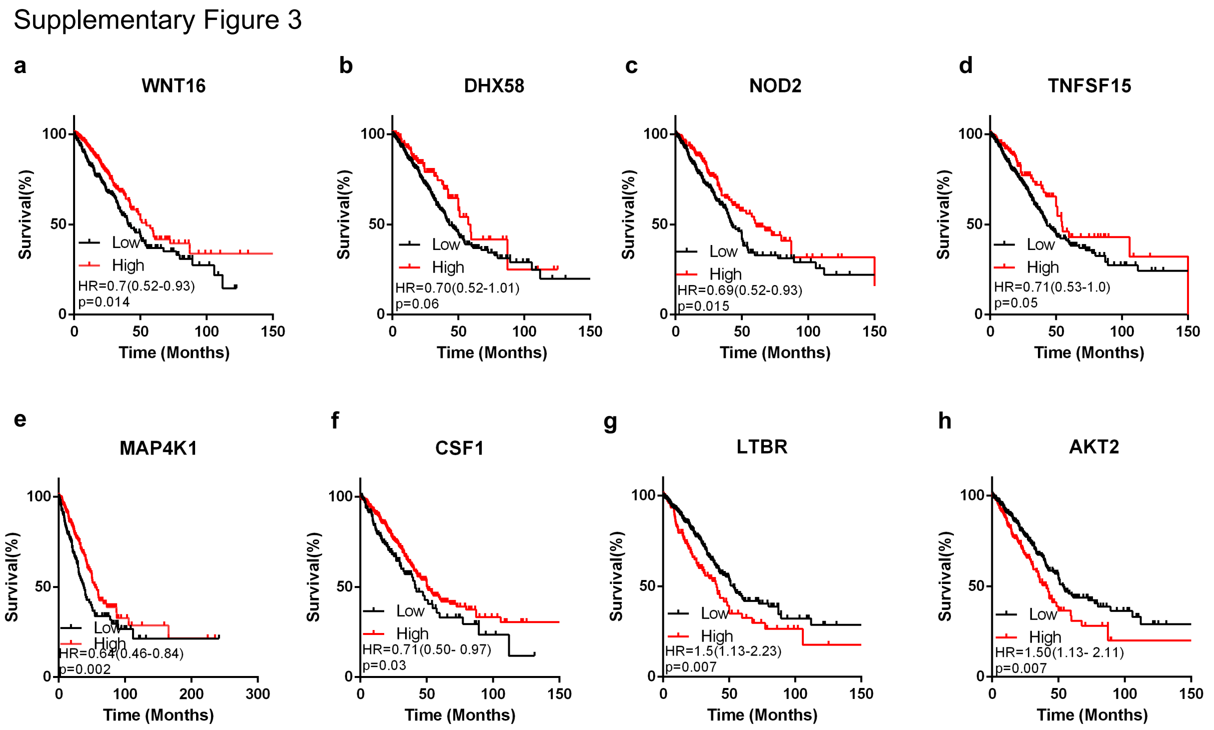

Supplementary Figure 3. Kaplan-Meier curves for overall survival rates associated with associated gene expression. **a** WNT16. **b** DHX58. **c** NOD2. **d** TNFSF15. **e** MAP4K1. **f** CSF1. **g** LTBR. **h** AKT2.

Supplementary Figure 4

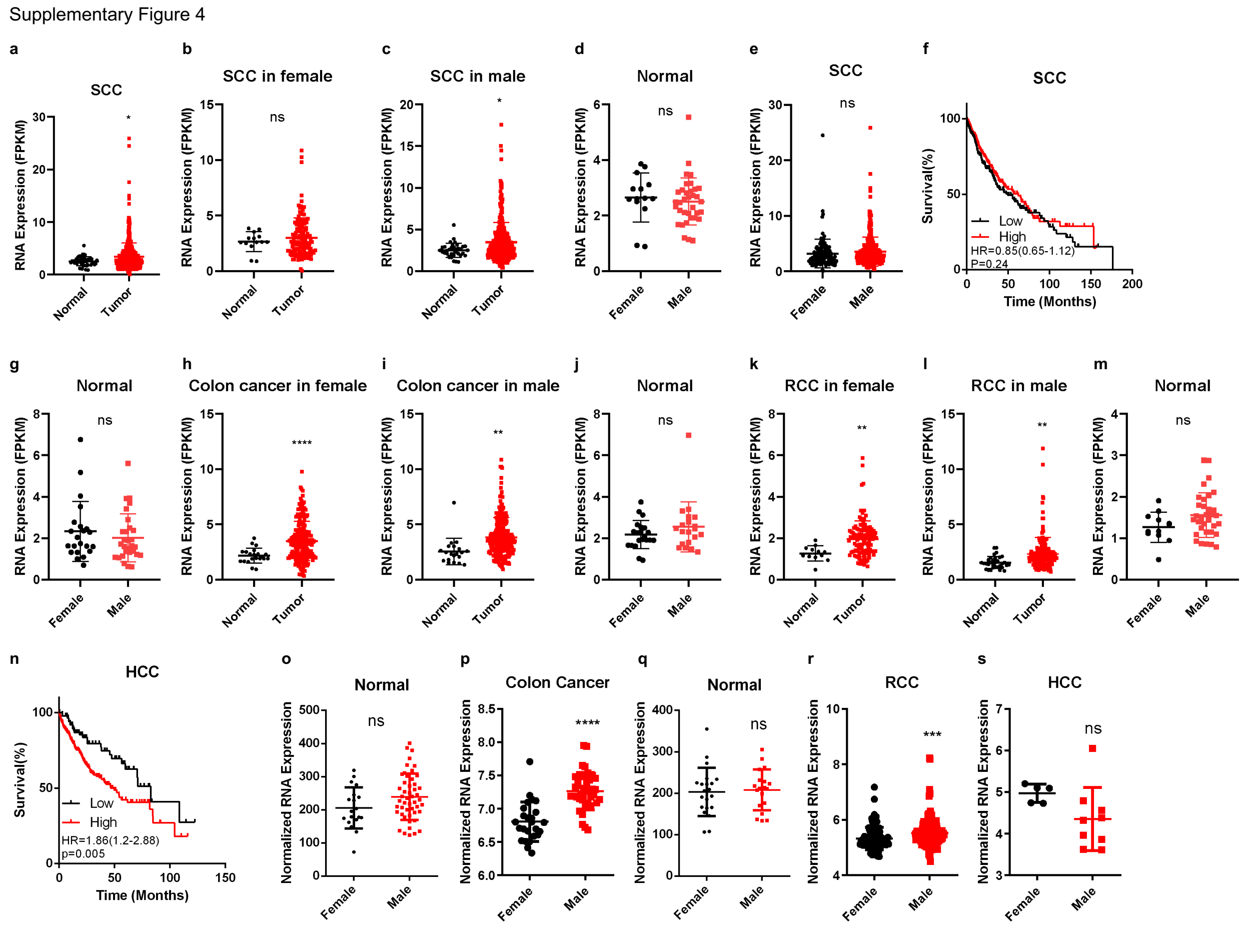

Supplementary Figure 4. The expression of LINC00263 in SCC, colon cancer, RCC and HCC. **a** the expression of LINC00263 is higher in 502 tumor tissue than 49 normal tissue in SCC (P=0.01). The different expression of LINC00263 between normal and tumor by sex in SCC (**b, c**), colon cancer **(h, i)** and RCC **(k, l)**. The expression of LINC00263 between females and males in normal tissue or tumor tissue in SCC (**d, e**), colon cancer **(g),** RCC **(j)** and HCC **(m)**. Data from the TCGA database. Kaplan-Meier curves for overall survival rates associated with LINC00263 expression in SCC (f) and HCC (n) **o, p** the expression of LINC00263 is higher in 40 male patients than 25 female patients in colon cancer (P<0.0001). Data from the GEO database (GSE29621). **q, r** the expression of LINC00263 is higher in 160 male patients than 102 female patients in RCC

(P=0.001). Data from the GEO database (GSE73731). **s** the expression of LINC00263 from the GEO database for hepatic carcinoma (GSE84402). There is no significant difference between 9 male patients and 5 female patients, P=0.1032. Data are shown as the mean ± SEM; n ≥ 3 independent experiments, two-tailed Student's t-test: ns nonsignificant (P > 0.05), *P <0.05, **P < 0.01, ***P < 0.001

Supplementary Figure 5

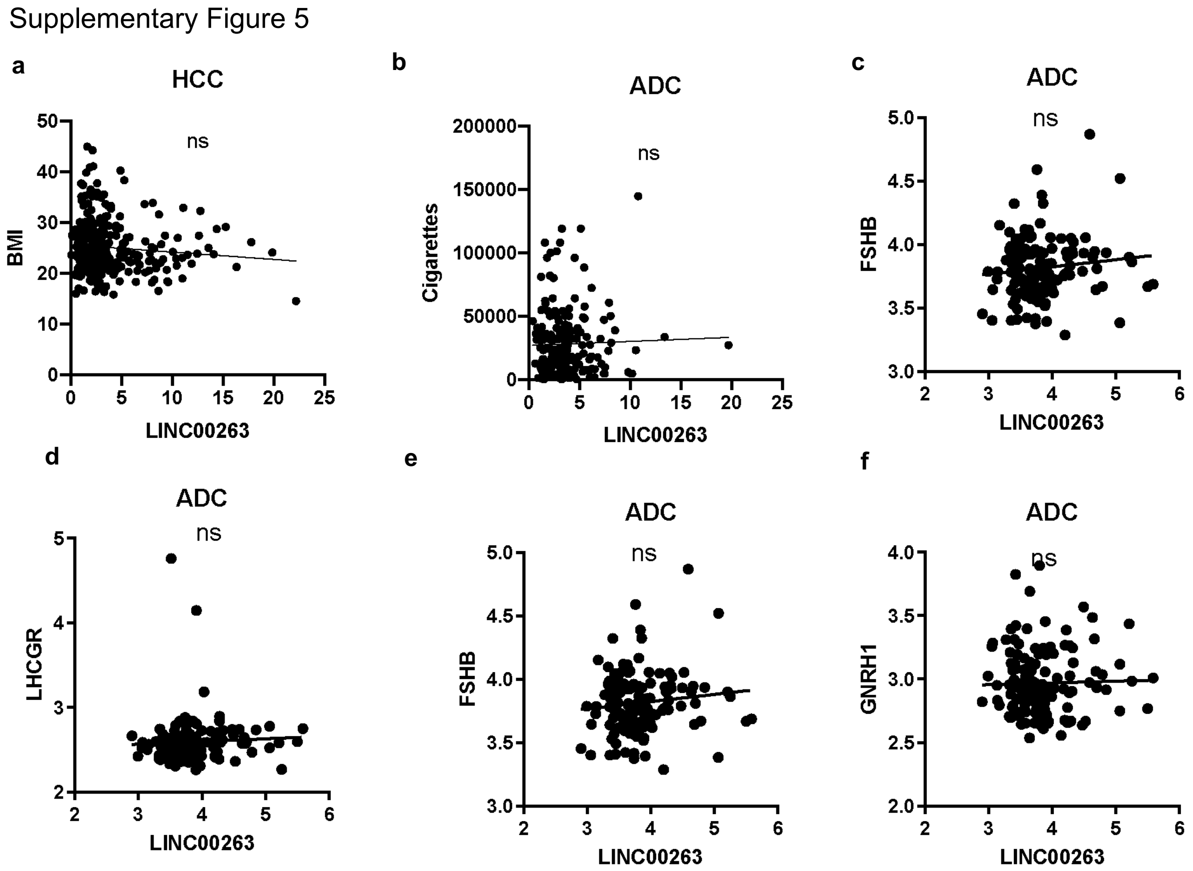

Supplementary Figure 5. The expression of LINC00263 has no significant correlation with BMI, cigarettes or the expression of PRL, LHCGR, FSHB, or GNRH1. Correlation between LINC00263 and BMI (**a**), cigarette counts (**b**), and the expression level of *PRL* (**c**) *LHCGR* (**d**) *FSHB* (**e**) and *GNRH1*(**f**) were analyzed.

Supplementary Figure 6

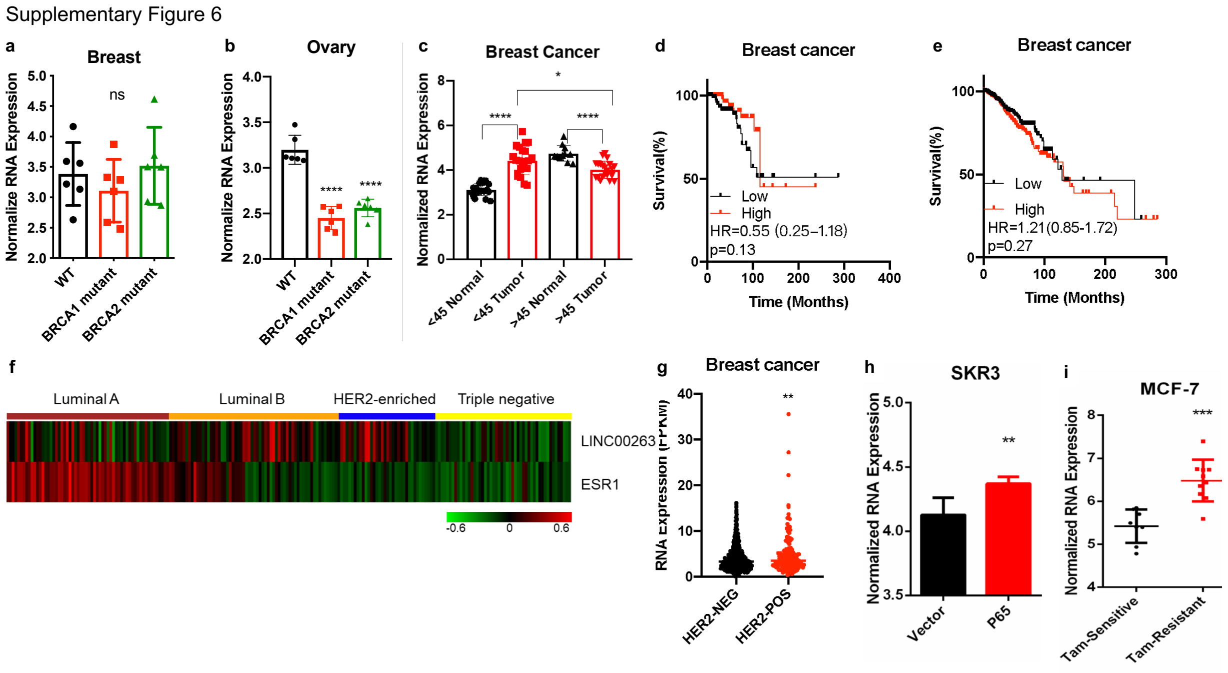

Supplementary Figure 6. The expression of LINC00263 in breast cancer, ovarian cancer, SKR3 cells and MCF-7 cells. The expression of LINC00263 in the WT patients, BRCA1 mutant patients and BRCA2 mutant patients from breast cancer (**a**) or ovarian cancer (**b**). Data from the GEO database (GSE19383). **c** Analysis of the expression of LINC00263 in breast cancer patients of different ages. Data from the GEO database (GSE29044). **d, e** Kaplan-Meier curves for overall survival rates associated with LINC00263 expression in younger than 45 years old breast cancer patients (**d**) and older (**e**). **f** the heatmap of LINC00263 and ESR1 in the different types of breast cancers. Data from the GEO database (GSE26639). **g** the expression of LINC00263 in HER-2 negative and HER-2 positive breast cancers. Data from the TCGA database. **h** analysis of the expression of LINC00263 in HER-2 positive cells with activated P65. Data from the GEO database (GSE52707). **i** comparison of the expression of LINC00263 between tamoxifen sensitive cells and

tamoxifen resistant cells. Data from the GEO database (GSE67916). Data are shown as the mean ± SEM; n ≥ 3 independent experiments, two-tailed Student's t-test: ns nonsignificant ($P > 0.05$), *$P < 0.05$, **$P < 0.01$, ***$P < 0.001$

Supplementary Figure 7

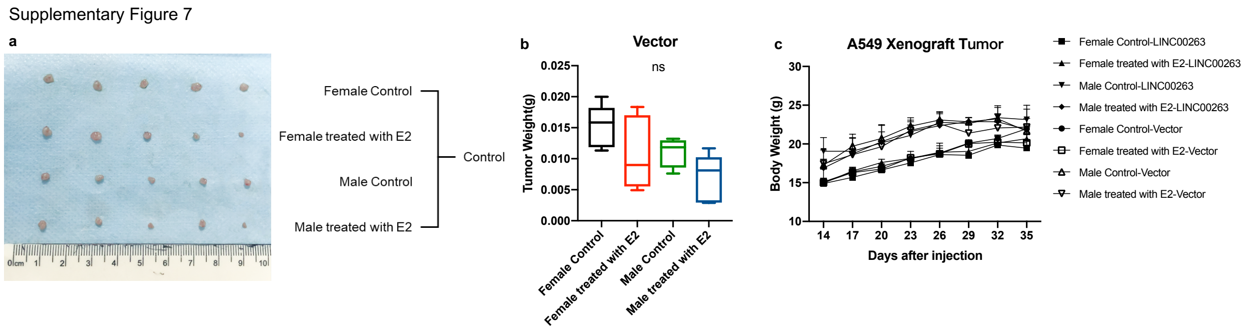

Supplementary Figure 7. Nude mice after the injection of A549 stably expressing the control vector expression are shown. Tumor formation was monitored at the indicated times; images (**a**), weights (**b**) and the weight change in nude mice (**c**) were recorded (n=8). Data are shown as the mean ± SEM; n ≥ 3 independent experiments, two-tailed Student's t-test: ns nonsignificant ($P > 0.05$), *$P < 0.05$, **$P < 0.01$, ***$P < 0.001$

Supplementary Figure 8.

Supplementary Figure 8

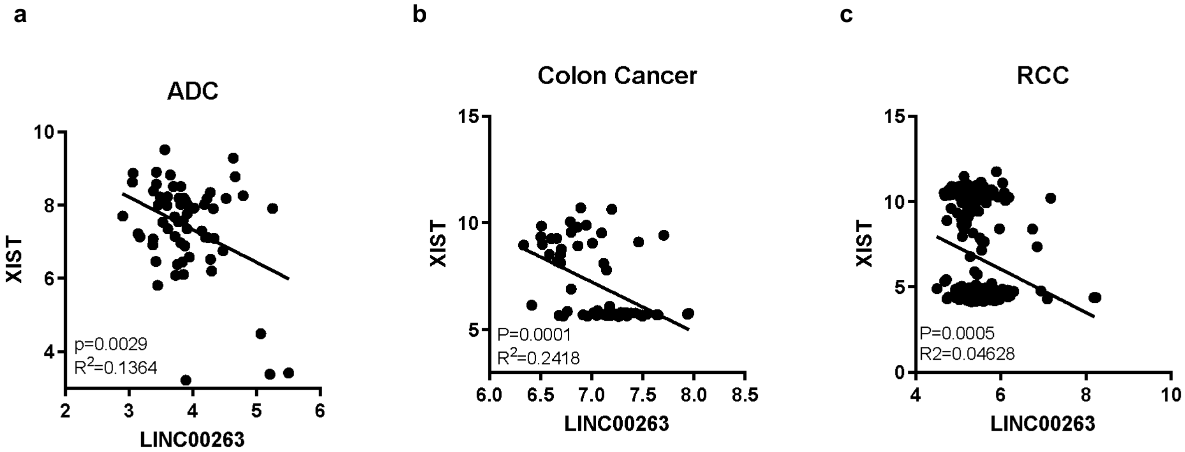

Supplementary Figure 8. The expression of LINC00263 is significantly negatively correlated with *XIST*. Correlation between *XIST* and LINC00263 in lung adenocarcinoma (**a**), colorectal cancer renal (**b**) and clear cell carcinoma (**c**).

Supplementary Table.1

GEO datasets used for research

Series	Platform	PMID	Relative Supplementary Figure
GSE14407	GPL570	20040092	Figure5s, Figure6a
GSE119087	GPL570	31091168	Figure6c
GSE11352	GPL570	17542648	Figure6d
GSE11428	GPL570	19632176	Figure6e
GSE7868	GPL570	17679089	Figure6f
GSE6364	GPL570	17510236	Figure6i
GSE11622	GPL570	18578861	Figure6j
GSE22600	GPL570	20959477	Figure6k, 6l
GSE26459	GPL570	21233418	Figure6m, 6n
GSE50081	GPL570	24305008	Supplementary Figure 2b
GSE29621	GPL570	22362069	Supplementary Figure 4o, 4p
GSE73731	GPL570	28779136	Supplementary Figure 4q, 4r
GSE84402	GPL570	28810927	Supplementary Figure 4s
GSE19383	GPL570	20051372	Supplementary Figure 6a, 6b
GSE29044	GPL570	23704896	Supplementary Figure 6c
GSE26639	GPL570	21631949	Supplementary Figure 6f
GSE52707	GPL570	24319068	Supplementary Figure 6h
GSE67916	GPL570	24882577	Supplementary Figure 6i
